# Association Between Internet Use and Body Dissatisfaction Among Young Females: Cross-Sectional Analysis of the Canadian Community Health Survey

**DOI:** 10.2196/jmir.5636

**Published:** 2017-02-09

**Authors:** Allison Carter, Jamie I Forrest, Angela Kaida

**Affiliations:** ^1^ Faculty of Health Sciences Simon Fraser University Burnaby, BC Canada; ^2^ Epidemiology and Population Health Program British Columbia Centre for Excellence in HIV/AIDS Vancouver, BC Canada; ^3^ School of Population and Public Health University of British Columbia Vancouver, BC Canada

**Keywords:** body satisfaction, Internet use, girls, young women, Canada

## Abstract

**Background:**

Recent research suggests Internet exposure, including Facebook use, is positively correlated with body dissatisfaction, especially among girls and young women. Canada has one of the highest Internet access rates in the world, yet no previous study has examined this relationship using nationally representative data.

**Objective:**

Our objective was to evaluate the relationship between Internet use and body dissatisfaction among a national, population-based sample of Canadian females 12-29 years of age.

**Methods:**

We used cross-sectional data from the Canadian Community Health Survey 2011-2012. Body dissatisfaction was measured using a 5-point Likert scale and defined as “very dissatisfied/dissatisfied” with one’s body. The explanatory variable was time spent using the Internet per week in the past 3 months, ranging from none/<1 hour to >20 hours. We used multinomial logistic regression to investigate whether greater Internet use was associated with increasing odds of being very dissatisfied/dissatisfied, neutral, or satisfied with one’s body, using very satisfied as the referent. Probability survey sampling weights were applied to all analyses.

**Results:**

Of 2983 included participants, sampled to represent 940,786 young Canadian females, most were 20-29 years old (61.98%) and living in households with an annual income Can $80,000 or more (44.61%). The prevalence of body dissatisfaction was 14.70%, and 25- to 29-year-olds were more likely than 12- to 14-year-olds to be very dissatisfied or dissatisfied with their body (20.76% vs 6.34%). Few (5.01%) reported none/<1 hour of Internet use, over half (56.93%) reported 1-10 hours, and one-fifth (19.52%) reported spending >20 hours online per week. Adjusting for age and income, the odds of being very dissatisfied/dissatisfied, relative to very satisfied, were greater in the highest versus lowest Internet use group (adjusted odds ratio [AOR] 3.03, 95% CI 1.19-7.70). The AORs for this level of body dissatisfaction increased across increasing levels of Internet use, ranging from 0.88 (95% CI 0.35-2.21) to 3.03 (95% CI 1.19-7.70). Additionally, those who spent 11-14 hours online were more likely to be neutral (AOR 3.66, 95% CI 1.17-11.45) and those who spent 15-20 hours online were more likely to be neutral (AOR 4.36, 95% CI 1.18-16.13) or satisfied (AOR 2.82, 95% CI 1.14-7.01) with their bodies, relative to very satisfied, compared with those spending no time or <1 hour online.

**Conclusions:**

A substantial proportion of Canadian females 12-29 years of age spent large amounts of time (>20 hours) on the Internet each week, and body dissatisfaction was significantly more likely among this group. Those who spent 11-20 hours online were also more likely to be less satisfied with their bodies. Efforts are needed to support girls and young women to achieve and maintain a positive body image in today’s digital age.

## Introduction

Body dissatisfaction, defined by a subjective negative view of one’s body weight and shape [1], is an important public health issue globally and in Canada, especially among girls and young women. An international survey of 11- to 15-year-old adolescents in 24 regions in Europe, the United States, and Canada found a high prevalence of body dissatisfaction across all countries, with variation seen by sex, age, and weight [2]. Among Canadians included in the study, 43% of girls (and 27% of boys) reported feeling dissatisfied with their body, and being older and overweight was positively correlated with body dissatisfaction [2]. In another longitudinal study of Canadian adolescents in Quebec, 57.1% of adolescent girls desired a thinner body at age 14 years, with that prevalence increasing to 65.8% at age 18 years; this is compared with 44.0% among adolescent boys at baseline, which remained unchanged 4 years later (39.5%; *P*=.17) [3]. Similarly high rates of body dissatisfaction are also seen among women during young adulthood, a time when social comparisons intensify [4,5]; research demonstrates that such comparisons, not just with peers but also with sociocultural images of thinness and attractiveness (eg, models and actors), can be detrimental to body image [6]. Understanding and mitigating risk factors for body dissatisfaction among females at an early age is critical given the high number of associated poor physical and mental health outcomes over the longer term, including low self-esteem [7], depression and suicidal ideation [8], disordered eating and exercise patterns [9], and “risky” sexual behaviors [10].

A large body of evidence suggests that sociocultural influences that equate female beauty with thinness play a powerful role in the development of body dissatisfaction among girls and young women [5,11,12]. Two reviews of experimental and correlational studies, including one meta-analysis, demonstrated that exposure to media such as television and magazines, which have ubiquitous images, messages, and advertisements promoting the ideal that women need to be thin, is associated with higher odds of body dissatisfaction [13,14]. More recently, interest has turned to the role of the Internet. Although young people use the Internet for multiple purposes, there is evidence that many of the websites and advertisements aimed at and accessed by girls and young women focus heavily on aspects of idealized female beauty (eg, health, fitness, fashion, makeup, and celebrities) [11]. Recent campaigns such as The Dove Campaign for Real Beauty [15] and Vancouver’s Raw Beauty Talks [16], which feature raw portraits of diverse women of varying sizes without makeup and filters, are attempting to alter this narrow picture of what beauty looks like. Nevertheless, the digital mainstream—coupled with the recent and rapid rise of social media platforms, such as Facebook and Instagram, that allow for greater appearance comparisons with people’s best photos of themselves (or “highlight reels”) [17]—presents tremendous challenges for women wanting to achieve or maintain a healthy body image. Indeed, recent research suggests that Internet exposure, including Facebook use [18-21], is positively correlated with body dissatisfaction.

Canada has one of the highest Internet access rates in the world, with over 85% of households having access to the Internet at home, and the average Internet user spending over 36 hours online every month, outside of work and school [22]. This is in contrast to roughly 40% of households in 2000 [23]. Additionally, it is estimated that 20 million Canadians now have a social networking account, with 18.5 million using Facebook [24], launched in 2004. For girls and young women in Canada, growing up in today’s digital age may exacerbate their risk for body dissatisfaction. However, to our knowledge, no previous study has evaluated the relationship between Internet use and body dissatisfaction in this population in Canada, nor used a nationally representative sample. Further, among the few international studies doing pioneering work in this area [18-21], none have examined a possible dose-response relationship. The primary purpose of this study was to answer the following research question: What is the relationship between amount of Internet use and body dissatisfaction among a national, population-based sample of Canadian female adolescents and young adults aged 12-29 years? The main hypothesis was that greater amounts of Internet use would be associated with increasing odds of body dissatisfaction.

## Methods

### Study Design

This study used data from the Public Use Microdata Files of the Canadian Community Health Survey (CCHS) 2011-2012 [25]. The CCHS is an annual population-based, cross-sectional survey conducted by Statistics Canada that collects information about general health status, personal health behaviors, use of health care services, and various social determinants of health for the purposes of health surveillance and population health research to improve the health of Canadians. The target population is individuals aged 12 years or older from all provinces, territories, and health regions residing in private households. Individuals working in the Canadian Armed Forces or those living in institutions, on First Nations’ reserves, or in certain remote areas of northern Ontario and Québec are excluded. Each annual CCHS cycle uses a complex, multistage sampling strategy to randomly select a sample of households over six 2-month collection periods, from January to December. Among sampled households, 1 individual is invited to participate in the survey, based on selection probabilities that consider age and household composition. The sampling strategy is also designed to oversample young people (12-19 years). Participants complete interviewer-administered, computer-assisted questionnaires, either in person or by telephone, that last approximately 60 minutes. A detailed description of the CCHS methodology is available from Statistics Canada [25].

### Study Population and Final Analytic Sample

**Figure 1:**
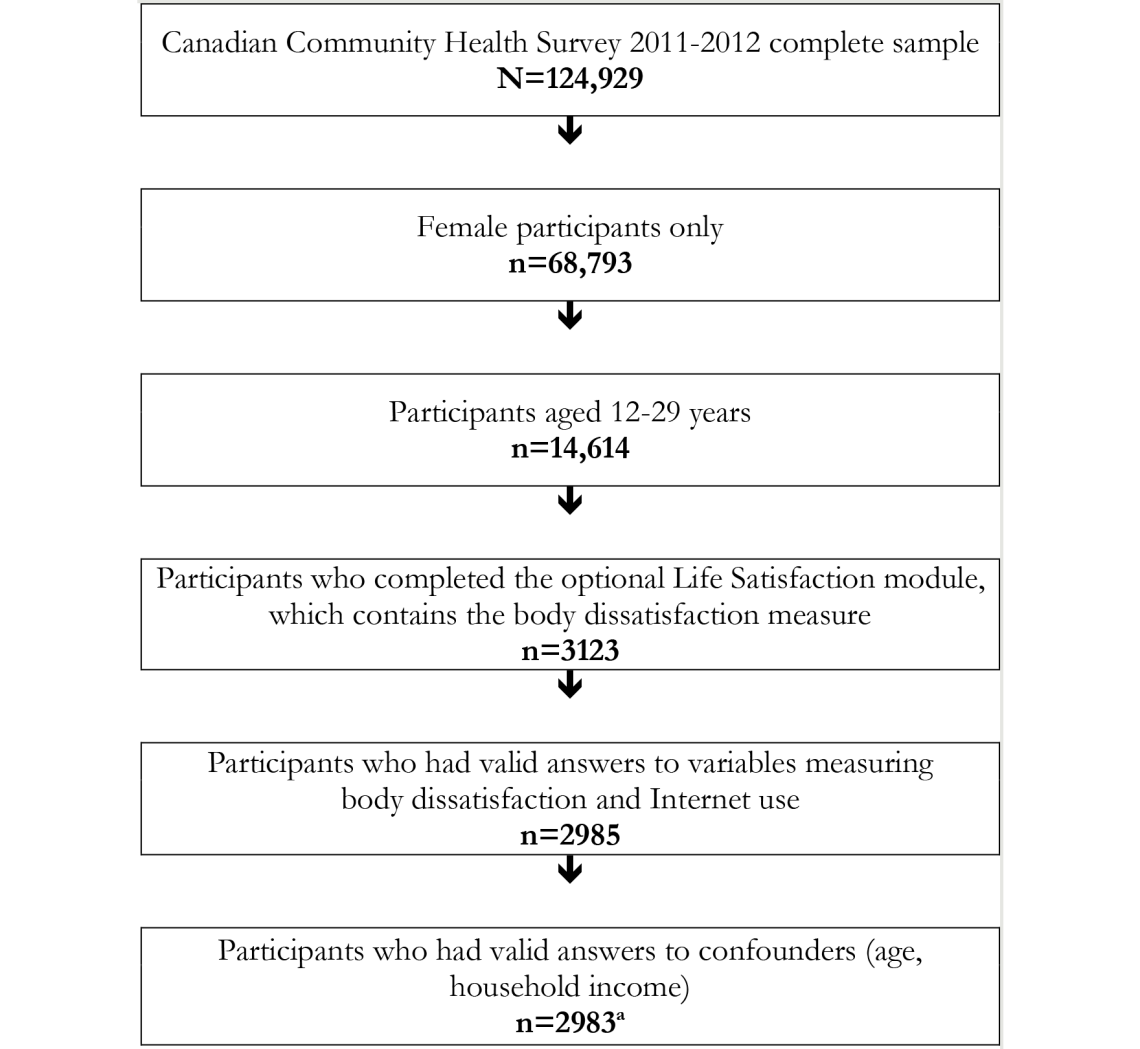
Flow chart of sample selection.^a^Final sample represents 940,786 Canadian adolescent girls and young women (weighted frequency).

In the CCHS 2011-2012, a total of 124,929 individuals participated out of 144,000 sampled households, representing a response rate of 86.76%. The overall sample was representative of 97% of the Canadian population aged 12 years or older. The participants included in the analysis for this study were female adolescents and young adults aged 12 to 29 years who reported valid responses to the primary outcome measure, explanatory variable, and confounders, described in the section below. As Figure 1 shows, 14,614 females aged 12 to 29 years participated in the CCHS 2011-2012. Of this total, 3123 were asked to complete the optional Satisfaction with Life module, which included perceptions of body dissatisfaction. Of these 3123 eligible adolescent girls and young women, we excluded an additional 230 participants due to invalid responses to the main study variables, such as “don’t know,” “prefer not to answer,” or “not asked/not stated” in the case of interviews completed by a proxy respondent (usually the parent for younger respondents). Thus, the final analytic sample included 2983 adolescent girls and young women. Using the survey weights provided by Statistics Canada, we assigned each person a weight to represent her contribution to the total population, taking into account the complex sampling design. With these survey weights applied, this sample represented 940,786 Canadians of this age and sex.

### Study Variables

The primary outcome was body dissatisfaction, measured using the following question: “How satisfied are you with the way your body looks?” Possible responses were “very satisfied,” “satisfied,” “neither satisfied nor dissatisfied,” “dissatisfied,” and “very dissatisfied.” We combined the responses “dissatisfied” and “very dissatisfied” due to low sample size and used them as the event of interest. The main explanatory variable was Internet use, assessed by the following proxy question: “In a typical week in the past 3 months, how much time did you usually spend on a computer, including playing computer games and using the Internet or World Wide Web?” Responses were “none or less than 1 hour,” “1-2 hours,” “3-5 hours,” “6-10 hours,” “11-14 hours,” “15-20 hours,” and “more than 20 hours.” This time frame does not include work or school, allowing for the estimation of leisure computer use, which is time spent largely online [22]. Nevertheless, this is likely a conservative estimate of Internet use, especially as it excludes mobile use (a major Internet source for this demographic) and includes computer games, which is discussed at length in the limitations section and highlights the challenge of using population-based data administered by national statistics agencies. Also, while this specific CCHS measure has not been previously validated, studies suggest that self-report measures are reliable tools to assess Internet use [26]. Confounders known to be determinants of body dissatisfaction and associated with Internet use [13,27] considered in the analyses were age (12-14, 15-17, 18-19, 20-24, and 25-29 years) and annual household income (in Can $: <20,000, 20,000-39,999, 40,000-59,999, 60,000-79,999, ≥80,000).

Other confounders we considered were ethnicity and body mass index (BMI). The CCHS categorizes ethnicity as white versus nonwhite according to a predetermined list without consideration of other meaningful dimensions of racial identity that may differentially affect body positivity. As such, we did not include ethnicity as a confounder in this analysis, and we feel that controlling for household income blocked some of the effects of ethnicity. Indeed, bivariable analyses revealed a strong association between these 2 variables, with increasing proportions of participants identifying as white, moving from the lowest to the higher income categories: 50.76%, 56.80%, 56.07%, 65.53%, and 74.17%, respectively. BMI was a strong predictor of body satisfaction, so much so that over 90.20% of those who were *not* overweight/obese were very satisfied with their bodies (n=468), with a small cell size in this outcome level for those who were overweight/obese (n=48). Inclusion of BMI in the adjusted multivariable model significantly increased the magnitude of the effect estimates of Internet use on body dissatisfaction and widened the variance around those estimates considerably. On one hand, this is an indicator of a strong confounder, but on another hand, it could be overadjustment, since BMI is highly correlated with body satisfaction. Importantly, the addition of BMI did not change the overall conclusions of the study. Further, in adding BMI, we lost 310 participants from the sample due to missing or invalid responses to this measure. Given these issues (eg, collinearity with body satisfaction, inflation of estimates and variance, and reduced sample size), as well as the limitations associated with the broad proxy measure of Internet use provided by the CCHS, we decided to take a conservative approach and exclude BMI from the final model, since it raised more issues regarding the statistical and analytical models than it solved conceptually.

### Analysis Plan

We calculated descriptive statistics using frequencies (n) and weighted percentages (%) to provide baseline characteristics, and the prevalence and patterns of body dissatisfaction and Internet use for the overall study sample. Bivariable analyses were conducted of the explanatory variable and confounders by the outcome measure. Associations were tested using Pearson chi-square test statistic. Binomial logistic regression [28] analyses were used to produce unadjusted estimates (crude odds ratios [ORs] and 95% CIs) of the association between body dissatisfaction and Internet use, as well as for the association between the confounders and the outcome. On review of the bivariable results, we used a multinomial logistic regression model [29], adjusting for confounders, to investigate whether greater amounts of Internet use were associated with increased odds of being very dissatisfied/dissatisfied, neutral, or satisfied with one’s body, using very satisfied as the referent, and report adjusted ORs (AORs) and 95% CIs.

For all models, age shifted the estimates by a significant amount (ie, >40%), while we observed only minor adjustments when adding household income to the model. Model fit statistics were better with income added (ie, lower Akaike information criterion) [30]; given this, and literature that suggests income may be an important confounder [27], we kept income in the model. Sensitivity analyses were also conducted to examine whether the number of variables in the model or an uneven distribution of cell sizes may have been contributing to the variability around the estimates; accordingly, we treated household income as a continuous measure and collapsed the 2 lowest and 2 highest categories of Internet use. The AORs and 95% CIs remained similar, and we observed a slightly poorer model fit (ie, higher Akaike information criterion); thus, we retained the original model.

All analyses were conducted using SAS version 9.3 for Mac (SAS Institute Inc). As mentioned, we weighted respondent data to account for the nonrandom sampling scheme and uneven probability of selection into the survey sample, using probability survey sampling weights developed and provided by Statistics Canada. These weights were applied to all analyses in order to produce more precise point estimates and variances (for details of the weighting procedures: [31]). Ethics approval for this study was covered under the publicly accessible data clause (Item 1.3.1) of the University of British Columbia’s Policy #89 on Research and Other Studies Involving Human Subjects.

## Results

### Baseline Characteristics

Of the 2983 adolescent girls and young women included in this study, most were 20 to 29 years of age (61.98%), versus less than 20 years, and lived in households where the annual income was Can $80,000 or greater (44.61%) (the highest level available for self-report in CCHS). Table 1 shows the baseline characteristics of this population as well as the patterns of Internet use and confounders by different levels of body dissatisfaction or satisfaction.

**Table 1:** Baseline characteristics and bivariable associations with body satisfaction and dissatisfaction among Canadian adolescent girls and young women aged 12-29 years: Canadian Community Health Survey 2011-2012 (n=2983).

Variables		Total n (%)^a^	Body satisfaction				
			Very dissatisfied/ dissatisfied (n=440, 14.70%) n (%)^a^	Neutral (n=349, 12.82%) n (%)^a^	Satisfied (n=1604, 53.44%) n (%)^a^	Very satisfied (n=590, 19.04%) n (%)^a^	*P* value
**Internet use (per week in the past 3 months), in hours**							<.001
	None/<1	159 (5.01)	33 (6.1)	14 (3.7)	79 (4.4)	33 (6.8)	
	1-2	529 (16.32)	64 (13.4)	63 (13.5)	270 (16.8)	132 (19.0)	
	3-5	556 (17.48)	61 (11.7)	64 (17.2)	324 (19.5)	107 (16.4)	
	6-10	749 (23.13)	98 (18.0)	80 (17.4)	415 (24.4)	156 (27.3)	
	11-14	358 (11.97)	53 (11.1)	46 (18.1)	194 (11.5)	65 (9.7)	
	15-20	167 (6.55)	33 (6.8)	21 (8.2)	86 (6.8)	27 (4.6)	
	>20	465 (19.52)	98 (33.0)	61 (21.9)	236 (16.4)	70 (16.3)	
**Age, in years**							<.001
	12-14	473 (13.24)	24 (5.7)	32 (9.4)	240 (11.8)	177 (25.6)	
	15-17	517 (15.38)	66 (13.6)	41 (10.5)	284 (15.6)	126 (19.3)	
	18-19	325 (9.40)	33 (7.6)	34 (8.5)	204 (10.2)	54 (9.1)	
	20-24	726 (30.68)	120 (28.9)	91 (37.0)	394 (30.7)	121 (27.7)	
	25-29	942 (31.30)	197 (44.2)	151 (34.5)	482 (31.6)	112 (18.3)	
**Annual household income, in Can $**							.09
	<20,000	210 (7.89)	33 (7.4)	26 (6.7)	113 (7.5)	38 (10.3)	
	20,000-39,999	505 (16.72)	83 (15.8)	52 (18.2)	272 (17.3)	98 (14.8)	
	40,000-59,999	487 (15.63)	80 (16.4)	63 (18.9)	268 (16.6)	76 (10.2)	
	60,000-79,999	471 (15.14)	68 (18.8)	68 (18.7)	255 (14.4)	80 (11.8)	
	≥80,000	1310 (44.61)	176 (41.7)	140 (37.3)	696 (44.2)	298 (52.9)	

^a^Column percentages are shown. Percentages have survey weights applied.

### Patterns of Internet Use and Confounders by Body Dissatisfaction

The overall prevalence of body dissatisfaction was 14.70%, and 25- to 29-year-olds were more likely than 12- to 14-year-olds to be very dissatisfied or dissatisfied with their body (20.76% versus 6.34%; data not shown). The majority of the sample reported satisfaction with their body at the satisfied (53.44%) or very satisfied level (19.04%). With respect to Internet use, we observed considerable variation in the time participants spent online in a typical week in the past 3 months: few (5.01%) reported none/less than 1 hour of Internet use, over half (56.93%) reported 1-10 hours, 18.52% reported 11-20 hours, and one-fifth (19.52%) reported more than 20 hours online per week. Examining Internet use by the outcome revealed that 33.0% of those who were very dissatisfied or dissatisfied with their body spent more than 20 hours online per week compared with only 16.3% of those who were very satisfied with their body (*P*<.001). In terms of age, among dissatisfied individuals, 44.2% were in the oldest age strata and 5.7% were in the youngest, while the pattern was reversed for very satisfied individuals (*P*<.001).

### Association Between Body Dissatisfaction and Internet Use

Table 2 presents the unadjusted ORs and AORs with 95% CIs of reporting increasing levels of body dissatisfaction by increasing amounts of Internet use. The unadjusted odds of being very dissatisfied or dissatisfied with one’s body, relative to very satisfied, were more than 2 times greater in the highest Internet use group (>20 hours) than in the lowest Internet use group (none/<1 hour) (OR 2.27, 95% CI 0.92-5.56). After adjusting for age and household income, the magnitude of the association between Internet use and body dissatisfaction was stronger (AOR 3.03, 95% CI 1.19-7.70), and the 95% CI excluded the null value of ‘1’ and reflected a range of effects, from moderate to more than a 7-fold increase in the odds of body dissatisfaction. It is noteworthy that the AORs for this outcome level of body dissatisfaction increased across increasing levels of Internet use, ranging from 0.88 (95% CI 0.35-2.21) to 3.03 (95% CI 1.19-7.70). The 95% CIs overlapped and included the null until the maximum level of Internet use; however, the gradual increase in point estimates is noteworthy and may suggest a possible dose-response relationship, in accordance with the principles of effect size estimation emphasizing CIs, as opposed to statistical significance testing [32]. For the 2 other outcome levels (neutral and satisfied), the strength of and variability around the adjusted effect estimates was similar, although we observed no pattern of increasing AORs. For instance, the adjusted odds of reporting being neutral, relative to very satisfied, were almost 4 times greater among those accessing the Internet for 11-14 hours than among those reporting no use or less than 1 hour of use (AOR 3.66, 95% CI 1.17-11.45), with even greater effects seen for those online for 15-20 hours (AOR 4.36, 95% CI 1.18-16.13). Lastly, with 2983 participants and using an alpha of .05, there was power greater than 90% to detect the final adjusted result for the association between body dissatisfaction and Internet use (≥20 hours versus none/<1 hour).

**Table 2:** Multinomial logistic regression results of being very dissatisfied/dissatisfied, neutral, or satisfied with one’s body in reference to very satisfied, as explained by increasing amounts of Internet use among Canadian adolescent girls and young women aged 12-29 years: Canadian Community Health Survey 2011-2012 (n=2983).

Internet use (per week in the past 3 months), in hours	Very dissatisfied/dissatisfied		Neutral		Satisfied	
	Unadjusted OR^a^ (95% CI)	Adjusted OR^b^ (95% CI)	Unadjusted OR (95% CI)	Adjusted OR^b^ (95% CI)	Unadjusted OR	Adjusted OR^b^
					(95% CI)	(95% CI)

None/<1	Reference	Reference	Reference	Reference	Reference	Reference
1-2	0.79 (0.33-1.91)	0.88 (0.35-2.21)	1.31 (0.46-3.73)	1.41 (0.48-4.19)	1.36 (0.67-2.76)	1.48 (0.71-3.11)
3-5	0.79 (0.32-1.98)	0.90 (0.34-2.36)	1.93 (0.66-5.66)	2.17 (0.72-6.60)	1.82 (0.87-3.79)	2.05 (0.94-4.47)
6-10	0.74 (0.31-1.72)	0.89 (0.36-2.21)	1.17 (0.42-3.31)	1.36 (0.46-3.99)	1.37 (0.68-2.75)	1.55 (0.74-3.26)
11-14	1.28 (0.52-3.18)	1.46 (0.55-3.84)	3.46 (1.13-10.59)	3.66 (1.17-11.45)^c^	1.83 (0.87-3.86)	1.95 (0.88-4.32)
15-20	1.66 (0.58-4.77)	2.51 (0.84-7.45)	3.34 (0.93-12.02)	4.36 (1.18-16.13)^c^	2.28 (0.93-5.60)	2.82 (1.14-7.01)^c^
>20 hours	2.27 (0.92-5.56)	3.03 (1.19-7.70)^c^	2.49 (0.82-7.47)	2.85 (0.92-8.81)	2.49 (0.82-7.47)	1.76 (0.80-3.86)

^a^OR: odds ratio.

^b^Adjusted for age and household income.

^c^AORs excluding the null.

## Discussion

### Principal Findings

In this national, population-based sample of Canadian females aged 12 to 29 years, 14.70% reported dissatisfaction with their bodies. A substantial proportion of participants spent large amounts of time (≥20 hours) on the Internet each week (19.52%), and body dissatisfaction was significantly more likely among this group. Further, those who spent 11-20 hours online were also more likely to be less satisfied with their bodies. While the data do not provide clear evidence of a dose-response relationship, these results may suggest a possible threshold effect. This is the first Canadian study to explore these patterns using nationally representative data, and these findings add to the international scientific research in this area.

The prevalence of body dissatisfaction in this study varied from estimates in other Canadian cohorts. A 2001/2002 study of 11-, 13-, and 15-year-old Canadian girls and adolescent girls found a much higher prevalence of body dissatisfaction (43.1%) [2], as did a 4-year longitudinal study of young women in Quebec (57.1% at baseline; 65.8% at follow-up) [3]. In another much larger study in Nova Scotia (n=2159), only 7.3% of girls in the sample reported body dissatisfaction [33]. Variations in the measures used may explain some of these differences. For instance, the latter study used “I like the way I look” as a proxy, coding girls responding with “never/almost never” as having body dissatisfaction and comparing them with all remaining girls reporting “sometimes/often/almost always.” The differences may also reflect differences in sample heterogeneity. Notably, the CCHS included a higher income sample, with nearly 60% of participants living in households with an annual income of at least Can $60,000; this is compared with 40% of participants in the Nova Scotia study. Additionally, Statistics Canada uses a complex sampling design to interview a random, representative sample of households across Canada. Thus, while it is unlikely that participants would be self-selected in the CCHS, this may, however, be a factor in other research using nonrepresentative samples.

The finding that high amounts of Internet use are associated with body dissatisfaction reflects previous research linking media exposure to women’s body image concerns. Two reviews reported moderate effect sizes for the relationship between exposure to multiple forms of media promoting the thin ideal and women’s body dissatisfaction [13,14]. To our knowledge, however, only a few other studies have specifically examined the impact of Internet use on body dissatisfaction [18-21]. This relationship was first investigated in a 2010 study of 156 Australian females aged 13-18 years [21], in which those authors found effects of Internet use on several body-related constructs, including internalization of thin ideals, appearance comparison, weight dissatisfaction, and drive for thinness. Interestingly, among the range of Internet sites examined in the Australian study, the most significant correlate of poor body image was time spent on Facebook. This study was repeated among a sample of preteenaged girls aged 10-12 years (n=189) [19], as well as a larger and more diverse cohort of 1087 adolescent girls aged 13-15 in Australia [18]. A similar finding was reported: Facebook use was more strongly correlated with poor body image compared with general Internet use. In 2015, Facebook was again linked to body dissatisfaction in a national sample of New Zealand women and men (n=11,017), across ages [20]. Interestingly, that study’s authors reported a cohort effect, with younger women who had grown up with more social media exposure reporting lower body satisfaction overall.

Our findings add to this literature, linking the amount of time spent using the Internet to body dissatisfaction and demonstrating an increasing likelihood of body dissatisfaction by increasing amounts of Internet use. The Internet has become ubiquitous in the lives of most Canadian young people, with continual accessibility being common, especially since the emergence of smartphones [22]. The specific content and platforms accessed online are likely wide in variety and remain unexplored in this study due to the lack of collection of such data in the CCHS. However, research suggests that girls and young women are highly exposed to online images and messages about female beauty [18-21], where the unattainable ideal is often someone who is “tall, young, usually white, has long, flowing hair, is surgically enhanced, blemish-free, and very thin” [34]. Social networking sites add to this milieu, creating a space for girls and young women to readily compare themselves with hundreds of peers on a daily basis. As body image experts highlight, “these comparisons are often made with at least somewhat idealized images (girls are likely to post only photographs showing themselves looking good or doing something ‘cool,’ and even these can be digitally altered)” [19]. Thus, in our study, we hypothesized that time spent online may increase exposure to these sociocultural forces that promote appearance as key to female worth. As previous research has demonstrated [19], this exposure may be related to the internalization of female beauty ideals, and, in turn, body dissatisfaction.

There is a need for future research to understand how the effect of Internet use may vary by age, sex, and time, among other meaningful characteristics. We conducted post hoc analyses to stimulate such efforts (tables not shown). In adjusted analyses with the entire sampled population (n=25,568), we observed a clearer dose-response relationship (with 4 of 6 AORs excluding the null and tighter 95% CIs: 1.29 (0.97-1.71), 1.28 (0.95-1.71), 1.41 (1.07-1.86), 1.78 (1.26-2.52), 2.46 (1.62-3.74), and 3.31 (2.34-4.69). Among those reporting body dissatisfaction, most were women (66.17%) and, of these, there was a roughly equal distribution of body dissatisfaction (18.84% to 22.26%) across these age strata: <30, 30-39, 40-49, 50-59, and ≥60 years. Thus, while individuals under 30 years of age used the Internet the most (accounting for 53% of those online ≥20 hours per week), sex-stratified inquiries into body dissatisfaction across all ages, not just young people, are needed. Next, we repeated analyses among adolescent boys and young men aged 12-29 years (n=2756). Relative to their female counterparts, body dissatisfaction was lower (6.08% vs 14.70%) and Internet use was higher (32.90% reported using it for ≥15 hours per week vs 26.07%). The adjusted odds of reporting being neutral (vs very satisfied) were 3.53 times greater among males accessing the Internet for 15-20 hours (vs none/<1 hour; 95% CI 1.17-10.69); the comparable best estimate for females was higher (4.36, 95% CI 1.18-16.13). All other effect estimates were not significant. These data may suggest sex differences in the link between Internet use and body dissatisfaction. Lastly, we examined data from 2003 (Cycle 2.1), the first year that questions about body satisfaction and Internet use were added to the CCHS and before the rise of smartphones and Facebook. Among females aged 12-29 (n=1565), the prevalence of body dissatisfaction was the same (14.77%) but Internet use was much lower (6.81% were online ≥15 hours per week vs 26.07% in 2011-2012). Adjusting for age and income, only 2 estimates were >2 and excluded the null, both comparing neutral in reference to very satisfied, including AOR 2.61 (95% CI 1.26-5.44) for those online 3-5 hours and AOR 4.61 (95% CI 1.23-17.26) for those online more than 20 hours. This should be explored further in longitudinal research with the same sample followed in time.

In addition to the analyses mentioned above, we conducted one further post hoc sensitivity analysis stratifying by BMI status to see whether the findings persisted across strata (ie, not overweight/obese vs overweight/obese). The sample sizes were extremely small, given 6 categories of Internet use by 4 categories of body satisfaction by 2 categories of weight, resulting in unstable models, estimates, and 95% CIs. However, the models did converge and provide some evidence of an interaction effect that should be investigated in future studies with sufficient sample size. Briefly, the findings for those not overweight/obese were consistent with those for the original model among the full sample—namely, the strongest effects were seen at the highest levels of Internet use (levels 5 through 7), and the magnitude of the effect varied by level of body satisfaction. The highest estimates were seen for neutral relative to very satisfied, although effect estimates were stronger in the normal BMI stratum, but the 95% CIs were very wide (eg, for the outcome of neutral, comparing 15-20 hours of Internet use vs none, the AOR was 4.36, 95% CI 1.18-16.13 in the original model and 12.98, 95% CI 2.22-76.00 in the not overweight/obese stratum). Thus, our conclusions are largely similar for this group. In contrast, 2 distinctive patterns were noteworthy among the stratum of overweight/obese individuals, although again we caution that the 95% CIs were extremely wide. First, the estimates were at their lowest for the neutral versus very satisfied comparisons across all levels of Internet use (eg, for 15-20 hours vs none, the AOR was 3.04, 95% CI 0.21-44.51, which is much smaller than the figures cited above), and the estimates were at their highest for the very dissatisfied/dissatisfied versus very satisfied comparisons across all levels of Internet use (eg, for ≥20 hours vs none, the AOR was 3.03, 95% CI 1.19-7.70 in the original model, 1.85, 95% CI 0.60-5.72 in the not overweight/obese stratum, and 8.82, 95% CI 0.86-90.33 in the overweight/obese stratum). Second, within *each* level of body satisfaction, we saw fairly high odds across *all* levels of Internet use, even 1-2 hours (eg, for the outcome of very dissatisfied/dissatisfied, comparing 1-2 hours of Internet use vs none, the AOR was 1.48, 95% CI 0.71-3.11 in the original model, 1.85, 95% CI 0.81-4.21 in the not overweight/obese stratum, and 6.49, 95% CI 1.06-39.66 in the overweight/obese stratum). The emergence of strong odds for dissatisfaction with body image across all levels of Internet use are consistent with what one might hypothesize for those who are overweight/obese. Thus, there is some evidence of an interaction effect that should be investigated in future studies, but this dataset is too underpowered to report on these findings as primary conclusions.

### Limitations

First, as mentioned, the measure of Internet use available in the CCHS was broad and lacked specificity, referring to time spent on a computer including playing computer games as well as surfing the “World Wide Web.” While computers are used for multiple purposes, many users spend their time online [22], and research suggests the most highly used online platform among girls and young women is Facebook [18], as well as magazine, celebrity, and beauty websites [11,18,19,21]. Further, a recent content analysis of video games found that these types of platforms perpetuate thin representations of female bodies [35]. Further, the exclusion of mobile use to assess Internet use is a strong limitation of this variable, since the Internet is accessed mainly via mobile devices today. Thus, it is likely that the measure used in this study produced a more conservative estimate of the relationship between Internet use and body dissatisfaction. This is a limitation of using population-based data administered by national statistics agencies. While we lack an in-depth validated measure of exposure, the study is strengthened by the use of a large, national, population-based dataset that provides exploration of relationships at the population-based level to inform the next studies. Future research, including forthcoming CCHS cycles, should contain more precise and nuanced measures of Internet use, including social media and mobile use.

Second, self-reporting of body dissatisfaction and Internet use to study interviewers may have been influenced by intentional or nonintentional social desirability bias, and in the case of Internet use, memory distortions. For instance, participants may have overreported their satisfaction level with their body. An underestimation of Internet use is also plausible, especially since figures suggest that many people under 30 are connected almost constantly or at least multiple times a day [36]. Because of this, measuring Internet use up to 20 hours a week could have created a ceiling effect, and interesting relationships between Internet use and body dissatisfaction may have been obscured through collapsing into a single category those people who use the Internet for 20 hours and those who use it almost constantly throughout their day. Both of these occurrences may have attenuated effects. Future research on this topic should consider using technology to track Internet use in real time.

Third, we derived the data from a cross-sectional survey, and one possible theoretical explanation for the findings is that women who start off with poor body satisfaction may be more likely to increase their Internet use. Prospective cohort studies that evaluate this relationship over time are needed.

Fourth, girls and young women are not a homogeneous group. They vary in age, sex (biological), gender (social), ethnicity, income, education, resiliency, and many other social identities and cultural factors (including Internet exposure), all of which can intersect in meaningful ways to influence the development of body dissatisfaction. Our primary focus was to measure the effect of Internet use (ie, we were not concerned with identifying the range of possible predictors). Future research should examine intersectional differences between multiple social categories. The strengths of this study are its large sample size and that it is based on a national population representative of 940,786 Canadian females aged 12-29 years.

### Conclusion

Given the number of poor health outcomes associated with body dissatisfaction [7-10], and the significant rise in Internet and social media use [22-24] that allows for unprecedented appearance comparisons [37], public health efforts are needed to support Canadian girls and young women to achieve and maintain a positive body image in today’s digital age. Evidence-based guidelines recommending healthier Internet use could be offered to empower girls and young women with the knowledge and skills to develop an authentic, healthy identity while engaging online. As activists and experts highlight [16,34], this may include limiting daily screen time, abstaining from using social media for 1 to 3 days, paying attention to the kind of content consumed and the impact it may be having on how we feel about ourselves, unfollowing people or sites that cause harmful self-evaluations, and, conversely, following those who promote health and well-being for ourselves. Further, public health education campaigns in schools and online, such as the “More to Her” media literacy curriculum by Raw Beauty Talks [16] or the 8-week Body Image Resilience Program by Beauty Redefined [34], are needed to promote confidence, self-love, and appreciation of oneself beyond what appears on the outside for all girls and women, in all of their diversity. Lastly, action is needed at policy levels to encourage advertisers and corporations to limit the editing of images and promote diverse and realistic representations of women. With Internet use only destined to grow, the time is now to reclaim definitions of female beauty and worth.

## Abbreviations

AOR: adjusted odds ratio
BMI: body mass index
CCHS: Canadian Community Health Survey
OR: odds ratio
